# Nutrients, Phytochemicals, and Antioxidant Capacity of Red Raspberry Nectar Fermented with *Lacticaseibacillus paracasei*

**DOI:** 10.3390/foods13223666

**Published:** 2024-11-18

**Authors:** Feng Shi, Yin Qin, Shuyi Qiu, You Luo

**Affiliations:** 1 School of Liquor and Food Engineering, Guizhou University, Guiyang 550025, China; shifeng2083@163.com (F.S.); 20130231@git.edu.cn (Y.Q.); syqiu@gzu.edu.cn (S.Q.); 2 School of Food and Drug Manufacturing Engineering, Guizhou Institute of Technology, Guiyang 550003, China; 3 Centre for Nutrition and Food Science, Queensland Alliance for Agriculture and Food Innovation, The University of Queensland, Brisbane, QLD 4068, Australia

**Keywords:** red raspberry nectar, fermentation, *Lacticaseibacillus paracasei*, phytochemical, metabolomics

## Abstract

Fresh raspberries are highly perishable, but lactic acid bacteria fermentation offers a favourable method for developing healthy products. This study investigated the effects of *Lacticaseibacillus paracasei* fermentation on the nutrients and phytochemicals of red raspberry nectar using widely targeted metabolomics, as well as its antioxidant activity. The fermentation notably disrupted the raspberry tissue structure, reshaped its non-volatile composition, and increased its DPPH and hydroxyl free radical scavenging abilities. A total of 261 compounds showed significant differences, with 198 upregulated and 63 downregulated. Among these, certain flavonoid glucosides (e.g., pelargonid-in-3-*O*-rutinoside, delphinidin-3-*O*-rutinoside-7-*O*-glucoside, and kaempferol-3-*O*-glucoside) were significantly downregulated, while some bioactive phenolic acids (e.g., 3-(4-Hydroxyphenyl)-propionic acid and DL-3-phenyllactic acid), alkaloids (e.g., deoxymutaaspergillic acid and indole-3-lactic acid), amino acids (e.g., L-phenylalanine and L-glutamine), and B vitamins (e.g., VB6, VB7, and VB3) were substantially upregulated. Furthermore, the Kyoto Encyclopedia of Genes and Genomes (KEGG) annotation and enrichment analysis revealed that metabolic pathways and the biosynthesis of secondary metabolites contributed significantly to the new profile of fermented red raspberry nectar. These findings provide valuable insights for developing fermented raspberry products using *Lacticaseibacillus paracasei*, which can help minimise fresh raspberry loss and enhance their valorisation.

## 1. Introduction

*Rubus idaeus* (red raspberry) is a highly nutritious fruit that provides a rich source of dietary fibre, vitamins, and phenolic compounds [1]. A growing body of studies have shown that red raspberries have positive effects on human health and wellness, such as anti-cancer [2] and anti-diabetic activity [3,4]. However, fresh red raspberries have an extremely short shelf life due to their delicate tissue structure and high respiration rate [5,6]. Freezing and frozen storage are popular methods for prolonging their quality and shelf life, while fermentation is a safer, more sustainable, and preferred strategy for preserving fresh foods and creating new delicious and healthy products [7].

Probiotic fermentation is a traditional and favourable processing method that can greatly enhance the flavour, nutritional profile, and health benefits of foods [7,8]. *Lactobacillus* species are considered safe starter cultures and probiotics which are widely used in the manufacture of fermented foods such as yogurt, cheese, and kimchi. Numerous studies have verified that lactobacilli are appropriate strains for fruit processing and contribute to nutrient availability and flavour enhancement, as well as health benefits [9,10,11,12,13]. For example, Xu et al. reported that four lactobacilli, including *Levilactobacillus brevis*, *Lactiplantibacillus plantarum*, *Lacticaseibacillus paracasei*, and *Limosilactobacillus fermentum*, improved the total flavonoid and phenolic levels, vitamin C content, and antioxidant capacity of *Citrus* juice [13]. Wen et al. indicated that *Lacticaseibacillus casei* fermentation increased the polyphenol, flavone, and exopolysaccharide levels in litchi juice, thereby enhancing its immunomodulatory effects and gut-microbiota-regulating activity [9].

Several studies have examined how *Saccharomyces* or non-*Saccharomyces* yeast fermentation affects the volatile [14,15] and non-volatile compounds [16,17,18] in red raspberry juice. However, no research has yet explored the impact of pure fermentation with a single *Lactobacillus* strain on the non-volatile composition of red raspberry nectar. Our previous work showed that *Lacticaseibacillus paracasei* FBKL 1.0328 (*Lc. paracasei* FBKL 1.0328) significantly transformed phytochemicals in *Rosa roxburghii* Tratt juice and enhanced its antioxidant activity [19]. Therefore, this study aimed to comprehensively investigate the impact of *Lc. paracasei* FBKL 1.0328 fermentation on the nutrients, and phytochemicals of red raspberry nectar using widely targeted metabolomics, as well as its antioxidant capacity. The findings underscore the beneficial effects of *Lc. paracasei* FBKL 1.0328 fermentation on red raspberry nectar, including the enhanced antioxidant capacity and elevated levels of certain bioactive phenolic acids and nutrients, providing a valuable reference for the development of novel functional red raspberry products.

## 2. Materials and Methods

### 2.1. Samples and Chemicals

Fresh red raspberries were purchased from a Walmart in Guiyang, Guizhou, China. Their place of origin was Menghai County, Xishuangbanna, Yunan, China. *Lacticaseibacillus paracasei* FBKL 1.0328 was isolated from a local fermented food Suantang and kept at the Fermentation Engineering Laboratory of Guizhou University.

Acetonitrile, methanol, and formic acid (chromatographic grade) were provided by Merck (Darmstadt, Germany). DPPH was purchased from Shanghai Aladdin Biochemical Technology Co., Ltd. (Shanghai, China). Other chemicals were obtained from Sinopharm Chemical Reagent Co., Ltd. (Beijing, China).

### 2.2. Fermentation of Red Raspberry Nectar

The average weight of each red raspberry fruit is approximately four grams. Fresh red raspberries (172.63 g) were mixed with distilled water (340 mL), then pressed and filtered through 80-mesh nylon cloth to obtain raspberry nectar. Raspberry nectar (30 mL per tube) was transferred to 50 mL centrifuge tubes and sterilised at 121 °C for 15 min in a high-pressure steam steriliser (Shenan, Shanghai, China).

*Lc. paracasei* FBKL 1.0328 was activated with fresh MRS (de Man, Rogosa, and Sharpe) broth. The stock culture (1 mL) was added to 100 mL of MRS broth (Bio-way, Shanghai, China) and incubated at 37 °C for 24 h. The bacterial cells were then obtained by centrifugation (4500× *g*, 10 min, 4 °C), washed twice with sterile water, and resuspended in sterile phosphate-buffered saline (PBS) to be used as the inoculum for the red raspberry nectar.

The red raspberry nectar without inoculation was used as the control and labeled UFR. The fermentation group (FR) was inoculated with 7.21 Log CFU/mL of activated *Lc. paracasei* FBKL 1.0328. All samples were incubated statically in a 37 °C anaerobic incubator (Hengzi, Shanghai, China) for 48 h. After fermentation, samples were centrifuged (LICHEN, Shanghai, China) at 4 °C for 10 min (6580× *g*). The supernatants were stored at −80 °C for analysis, while the residues were freeze-dried for scanning electron microscopy (SEM) analysis.

### 2.3. Determination of Viable Cells

Viable cell counts were determined by the agar serial dilution method. The fermented red raspberry nectar (1 mL) was diluted with normal saline, and 100 μL of each dilution was plated on MRS agar. Samples were cultured at 37 °C for 48 h. The Petri dishes containing 30–300 colonies were counted and recorded as log CFU/mL [19].

### 2.4. Scanning Electron Microscope of UFR and FR Residues

A small amount of sample (unfermented/fermented nectar sediments) was adhered to a conductive adhesive and coated with gold/palladium alloy by the Oxford Quorum SC7620 sputter coating (Laughton, UK). The morphology of sample was collected by a ZEISS GeminiSEM 300 scanning electron microscope (Oberkochen, Germany) in secondary electron imaging mode.

### 2.5. Determination of Physicochemical Parameters of UFR and FR

The L (lightness), a (green/red), and b (blue/yellow) of samples were measured by a Chroma meter CR-400 (KONICA MINOLTA, Tokyo, Japan). The Brix (total soluble solids) of samples was measured with a handheld refractometer at 25 °C (VWR, Radnor, PA, USA). The total sugar and reducing sugar content of samples were measured by the phenol-sulfuric acid method [20] and the 3,5-dinitrosalicylic acid method [21], respectively. The pH values of samples were measured with a pH meter (PHS-2F, INESA, Shanghai, China). The total acidity was determined by titration with 0.1 mol/L of NaOH (National Standard of the people’s Republic of China, GB-12456-2021) [22]. Tartaric acid served as the reference acid for titration.

### 2.6. Determination of Total Phenolic and Total Flavonoid Content

The total phenolic content and total flavonoid content of the unfermented and fermented samples were determined by Folin–Ciocalteu and AlCl_3_ colorimetric method, respectively. The procedures followed those outlined in a previous study [19].

### 2.7. Identification of Non-Volatile Compounds

The non-volatile compounds of samples were detected and identified using an ultra-high-performance liquid chromatography–triple quadrupole mass spectrometry system, according to a method described by Li et al. [23]. Thawed samples were vortexed for 10 s. Subsequently, each sample (100 μL) was mixed with 100 μL of 70% methanol containing the internal standard (2-Chloro-L-phenylalanine, 1 ppm). After agitation for 15 min, samples were centrifuged at 4 °C for 3 min (14,800× *g*), and the supernatants were analysed by UHPLC (ExionLC^TM^ AD, Framingham, MA, USA) coupled with MS/MS (QTRAP 4500, SCIEX, Framingham, MA, USA).

The UHPLC conditions were as follows: column—Agilent SB-C18 (2.1 × 100 mm, 1.8 μm; Santa Clara, CA, USA), mobile phases—purified water containing 0.1% formic acid (A) and acetonitrile containing 0.1% (*v*/*v*) formic acid (B), flow rate—0.35 mL/min, injection volume—2 μL, column temperature—40 °C, and the elution gradient—0 min, 5% B; 0–9 min, 5–95% B; 9–10 min, 95% B; 10.00–11.10 min, 5% B; and 11.10–14 min, 5% B. The mass spectrometer was run in negative (−4500 V)/positive (5500 V) ionisation mode over a scan range of 100–1250 Da with the following settings: electrospray ionisation source temperature, 550 °C; ion source gas I, 50 psi; gas II, 60 psi; curtain gas, 25 psi; the collision-induced ionisation, high; the collision gas (nitrogen), medium; and the triple quadrupole mass spectrometer scan mode, Multiple Reaction Monitoring (MRM). Quality control (QC) samples were regularly tested to ensure method and instrument performance.

Compound identification was performed using MS/MS data, internal standards, the MS/MS library, and the Metware Database. Isotope signals, redundant signals containing K^+^, Na^+^, and NH_4_^+^ ions, and fragment ion signals from larger molecules were excluded during analysis. Compound quantification was achieved using the MRM mode on a triple quadrupole mass spectrometer. The precursor ion of the target compound was selected to minimise interferences, then fragmented in the collision cell to generate product ions. The triple quadrupole filtered these to select a specific characteristic fragment ion. After obtaining mass spectrometry data, peak area integration was performed for all chromatographic peaks, and the peak areas for the same metabolite across different samples were corrected and integrated.

### 2.8. Evaluation of In Vitro Antioxidant Activity

The antioxidant activity of samples was evaluated by DPPH, ABTS, and OH radical scavenging assays [24,25], as well as the reducing power test [26].

### 2.9. Statistical Analysis

All tests were performed in triplicate, and results were expressed as mean ± standard deviation. Significant differences between UFR and FR were determined by the paired *t*-test using SPSS 22.0 (IBM), with *p* < 0.05 considered statistically significant. Compound data were processed using hierarchical cluster analysis (HCA), principal component analysis (PCA), and orthonormal partial least-squares discriminant analysis (OPLS-DA) with the R package Complex Heatmap, R function “prcomp”, and MetaboAnalystR, respectively. Identified compounds were annotated through the Kyoto Encyclopedia of Genes and Genomes (KEGG) compound database.

## 3. Results and Discussion

### 3.1. The Effects of Lc. paracasei Fermentation on Red Raspberry Tissue Structure

**Figure 1:**
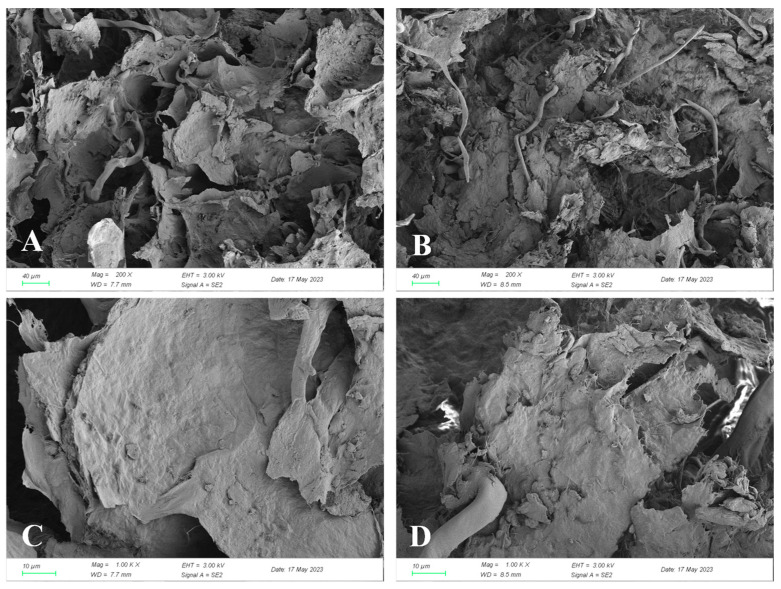
SEM morphology of UFR and FR tissue: UFR sample (**A**,**C**), and FR sample (**B**,**D**). UFR: unfermented red raspberry nectar, FR: fermented red raspberry nectar.

The surface morphology of unfermented and fermented red raspberry tissue is shown in Figure 1. The unfermented sample had a relatively smooth surface with numerous thin, intact laminae and a compact microstructure (Figure 1A,C), whereas the fermented sample exhibited a rough, fluffy surface with exposed filaments and scattered irregular fragments (Figure 1B,D). This evidence suggests that *Lc. paracasei* can considerably break down the structure of red raspberry tissue, facilitating the liberation and conversion of components. Similarly, Zhang et al. observed cracks and small fragments on the outer wall of bee pollen after fermentation [27]. The degradation of plant cell walls is driven by various enzymes, such as cellulase and pectinase, which enhances the bio-accessibility and bioavailability of nutrients and bioactive compounds in substrates [10,16,28].

### 3.2. The Viability of Lc. paracasei FBKL 1.0328 in Red Raspberry Nectar

As shown in Table 1, the viable cells of *Lc. paracasei* increased significantly from 7.21 log CFU/mL to 9.84 log CFU/mL after 48 h of fermentation (*p* < 0.05), indicating its ability to grow and proliferate in the red raspberry nectar without additional nutrients. This suggests *Lc. paracasei* is a promising strain for developing new raspberry products. Likewise, three LAB strains-*L. plantarum* NCU116, *L. acidophilus* NCU402, and *L. casei* NCU215-achieved robust growth in blueberry juice, reaching 8.0 log CFU/mL after 24 h of fermentation [12].

### 3.3. The Basic Physicochemical Properties of UFR and FR

*Lc. paracasei* fermentation resulted in a significant decrease in the L* value, a slight increase in the a* value, and a slight decrease in the b* value of red raspberry nectar, indicating reduced lightness and yellowness, but a redder hue (Table 1). The colour of the juice or nectar correlates with the pH and anthocyanin composition. Fermentation causes fluctuations in the pH and alters the anthocyanin levels, leading to changes in colour [12].

The total soluble solids of red raspberry nectar significantly dropped from 6.10 to 5.83 after fermentation. The total sugar content mildly decreased from 463.38 to 458.59 μg/mL, while the reducing sugar content slightly increased from 278.62 to 297.63 μg/mL (Table 1). This may be attributed to the hydrolysis of polysaccharides or glycosylated compounds. Sugars released from these complex compounds can partially compensate the consumed monosaccharides and disaccharides [29,30,31]. In addition to carbohydrates, *Lc. paracasei* can utilise other components to survive and thrive, such as protein, lipid, and organic acid. The variation in the sugar content during the LAB fermentation of fruits varied from case to case. For example, LAB fermentation significantly reduced the total soluble solids and glucose in blueberry juice [12]. A notable decline occurred in the sucrose concentration of a vegetable–fruit beverage after fermentation, while its total sugar content showed no significant change [32]. The total sugar content in the sea buckthorn juice was not significantly affected by the fermentation process, because the employed *L. plantarum* preferentially utilised organic acids instead of sugars as the carbon source [33]. The total acidity of red raspberry nectar notably increased from 9.92 to 12.24 g/L after fermentation. Correspondingly, the pH significantly decreased from 3.25 to 3.12 (Table 1). In general, LAB converts the sugars in substrates into organic acids, leading to a rise in the total acidity and a drop in the sugar content.

### 3.4. The Total Phenolic and Flavonoid Content of UFR and FR

Microbial fermentation can release and transform phenolics, thereby affecting their levels in foods. The total phenolic content and total flavonoid content in UFR were 346.39 μg GAE/mL and 482.28 μg RE/mL, respectively, while the total phenolic content and total flavonoid content in FR were 336.50 μg GAE/mL and 446.84 μg RE/mL, respectively (Table 1). *Lc. paracasei* fermentation did not significantly change the total phenolic content of red raspberry nectar but remarkably reduced its flavonoid content by 7.35%. Studies show that LAB fermentation either increased or decreased the total phenolic and flavonoid content of substrates. Some research indicates that LAB can boost the levels of phenolics and flavonoids [19,34,35]. Conversely, some studies report reductions in the total phenolic and flavonoid content after fermentation [11,36]. For example, Adebo et al. observed decreased flavonoid, phenolic, and tannin levels in *ting* (a Southern African food) fermented with *Lacticaseibacillus* strains [36]. Li et al. found a significant increase in the total phenolic content but a considerable decrease in the total flavonoid content of jujube juice after LAB fermentation [10]. These variations highlight that the effects of LAB fermentation on the polyphenolic concentration depends on the specific strains and substrates.

### 3.5. Overview of the Non-Volatile Composition Profile of UFR and FR

The non-volatile compounds in the unfermented and fermented red raspberry nectar were analysed qualitatively and quantitatively using UHPLC-MS/MS in the MRM model. The total ion current (TIC) chromatogram is shown in Supplementary Figure S1. The high overlap between samples and QC samples indicated the reproducibility, stability, and reliability of the data. Furthermore, the proportion of substances with coefficient of variation (CV) values below 0.3 in QC samples exceeded 85% (shown in Supplementary Figure S2), indicating that the experimental data were highly stable.

**Figure 2:**
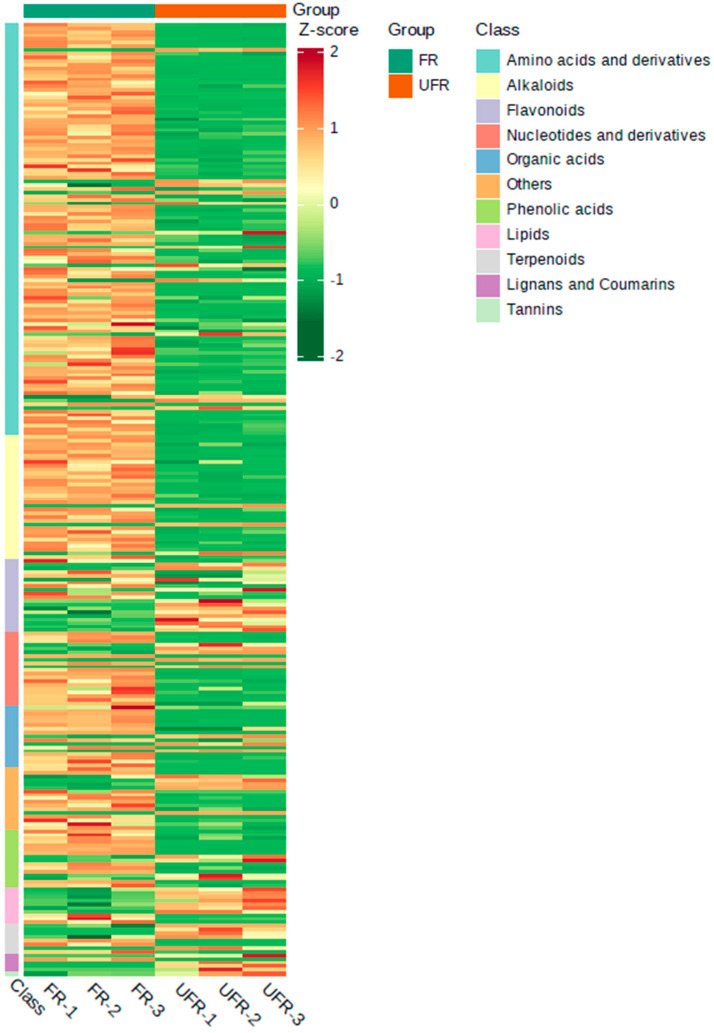
Heatmap for identified chemical composition of FR and UFR. UFR: unfermented red raspberry nectar, FR: fermented red raspberry nectar (Note: The columns represent the two groups, and each row indicates a compound in all samples; a color-coded scale grading from red to green means the relative content of metabolites from high to low).

A total of 1729 compounds were identified, which consisted of amino acids and their derivatives (20.53%, *n* = 355), phenolic acids (12.20%, *n* = 211), flavonoids (11.05%, *n* = 191), alkaloids (9.66%, *n* = 167), terpenoids (8.27%, *n* = 143), lignans and coumarins (6.13%, *n* = 106), lipids (5.73%, *n* = 99), organic acids (5.67%, *n* = 98), nucleotides and their derivatives (3.7%, *n* = 64), tannins (0.93%, *n* = 16), quinones (0.58%, *n* = 10), steroids (0.12%, *n* = 2), and others (15.44%, *n* = 267). A heatmap (Figure 2) visualises the relative content of these components, showing the distinct clustering of FR and UFR. Most compounds in FR, especially amino acids, their derivatives, and alkaloids, were significantly upregulated, highlighted in red. This coincided with previous findings where most amino acids and their derivatives and alkaloids in *Rosa roxburghii* Tratt juice were considerably upregulated after *Lc. paracasei* fermentation [19].

### 3.6. The Differences in Primary Nutrients and Phytochemicals Between UFR and FR

**Figure 3:**
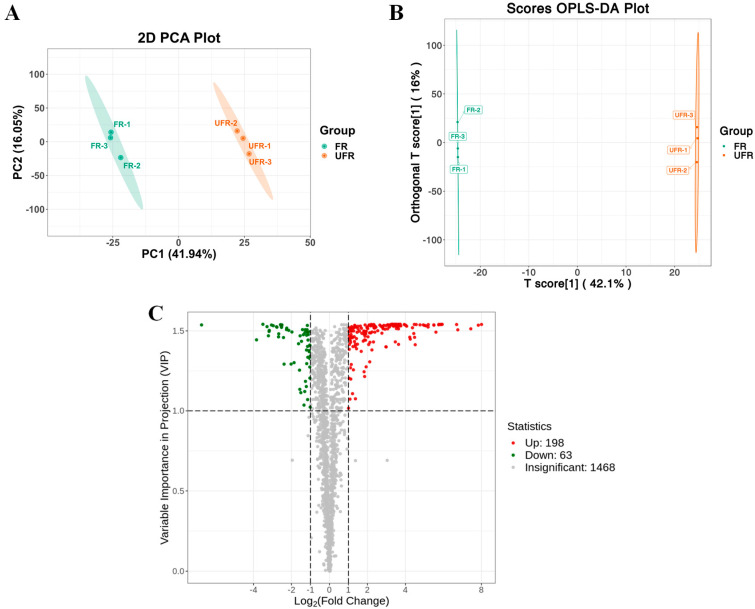

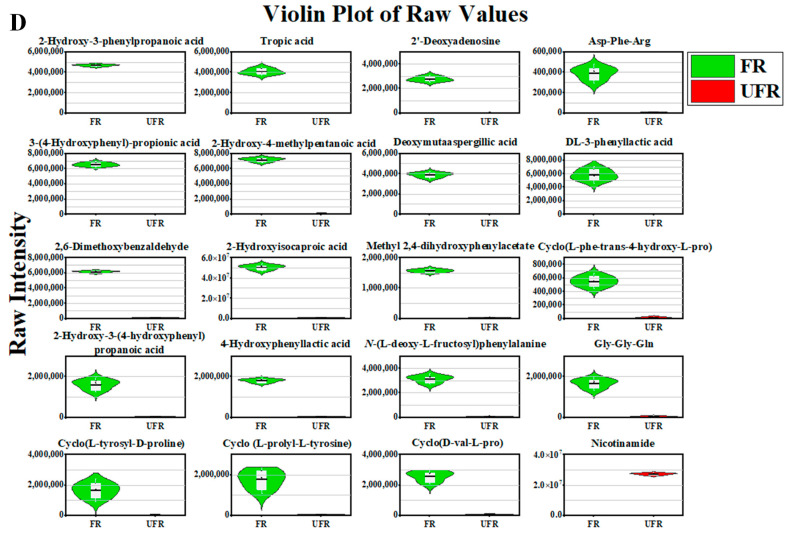
(**A**) PCA plot of all identified compounds of FR and UFR; (**B**) OPLS-DA plot of FR and UFR; (**C**) volcano plot of all identified compounds of FR and UFR; and (**D**) the violin plots of raw intensity of the top 20 differential compounds between FR and UFR. UFR: unfermented red raspberry nectar, FR: fermented red raspberry nectar.

*Lc. paracasei* fermentation remarkably transformed the chemical composition of red raspberry nectar. PCA analysis (Figure 3A) displayed a clear separation between FR and UFR, which was further confirmed by an OPLS-DA analysis (Figure 3B). A total of 261 compounds showed significant differences, with 198 upregulated and 63 downregulated (Figure 3C). This was consistent with some previous studies, in which lactic acid bacteria impacted the non-volatile and volatile compounds of fruit juice or nectar considerably [12,19,37].

The significantly differential compounds (n = 261) were classified into 11 categories, including amino acids and their derivatives (n = 113), alkaloids (n = 34), nucleotides and their derivatives (n = 20), flavonoids (n = 20), phenolic acids (n = 16), organic acids (n = 17), lipids (n = 10), terpenoids (n = 8), lignans and coumarins (n = 5), tannins (n = 1), and others (n = 17). Amino acids and their derivatives, phenolic compounds, and alkaloids were the predominant differential metabolites. The top 50 differential compounds between FR and UFR shown in Table 2 were screened by Variable Importance in Projection (VIP), VIP > 1, and Fold Change (FC), FC ≥ 2 or FC ≤ 0.5, based on the OPLS-DA model. Moreover, violin plots (Figure 3D) illustrate the relative content and distribution of the top 20 differential compounds between the FR and UFR group. Except for nicotinamide, all other compounds were remarkably upregulated in FR. The most prominently upregulated compounds, which were phenolic acids with a high abundance, include 3-(4-hydroxyphenyl)-propionic acid, DL-3-phenyllactic acid, 2-hydroxy-3-phenylpropanoic acid, and tropic acid. Additionally, amino acids and their derivatives were substantially upregulated, including Asp-Phe-Arg, Gly-Gly-Gln, Cyclo (L-prolyl-L-tyrosine), Cyclo(L-tyrosyl-D-proline), Cyclo(D-Val-L-Pro), Cyclo(L-Phe-trans-4-hydroxy-L-Pro), and *N*-(1-deoxy-1-fructosyl) phenylalanine.

#### 3.6.1. Amino Acids and Their Derivatives

A total of 113 amino acids and their derivatives were identified as differential metabolites between FR and UFR, with 100 upregulated and 13 downregulated. These compounds were mainly short peptides, cyclic peptides, and amino acid derivatives, which impact sensory quality and biological activities. For instance, *N*-(1-deoxy-1-fructosyl)leucine, a sweet amino acid derivative [38], increased by 16.12 times after fermentation. Essential amino acids such as L-phenylalanine (5.04-fold increase), DL-methionine (3.09-fold increase), and L-glutamine (2.62-fold increase) could influence the taste of raspberry nectar. Generally, glutamine provides a sweet taste, while phenylalanine, methionine, tryptophan, lysine, and isoleucine contribute bitterness. In addition, some upregulated cyclodipeptides, such as cyclo(Pro-Val), cyclo(Pro-Leu), and cyclo(L-Pro-L-Tyr), have shown antibacterial activity [39]. Different strains produce distinct amino acid profiles. The co-fermentation with *Lacticaseibacillus rhamnosus* and *Gluconacetobacter xylinus* reduced the content of bitter and sweet free amino acids in yacon–litchi–longan juice [40]. *W. anomalus* fermentation significantly decreased the bitter leucine content in black garlic juice [41].

#### 3.6.2. Organic Acids

Organic acids lower the pH of the fermenting “must” to a level where many spoilage bacteria cannot survive. On the other hand, they play an important role in the taste and flavour of fermented foods. *Lc. paracasei* fermentation modified the organic acid profile of red raspberry nectar, with 17 organic acids (14 upregulated and 3 downregulated) showing significant differences between FR and UFR. Notably, the L-lactic acid content substantially increased by 13.29 times due to the conversion of carbohydrates and malic acid during fermentation. Fumaric acid, L-malic acid, and D-malic acid were downregulated, leading to the reduced tartness of red raspberry nectar [42,43], while lactic acid and malic acid in Goji berry juice were significantly elevated after fermentation with Tibetan kefir grains containing lactic acid bacteria, acetic acid bacteria, and yeasts [44]. Additionally, the concentration of L-tartaric acid and oxalic acid in FR increased significantly by 15.80 and 13.07 times, respectively. Similar results were observed in *Limosilactobacillus reuteri* fermented apple juice [42]. But Zhao et al. found that LAB greatly decreased the tartaric acid and oxalic acid content in jujube–wolfberry juice [45]. These findings indicate that the strain-specific metabolism and substrates together create the organic acid profile of a product.

#### 3.6.3. Nucleotides and Their Derivatives

The fermentation significantly changed 20 nucleotides and their derivatives in red raspberry nectar, with 15 upregulated (such as 2′-deoxyadenosine, inosine, and thymine) and 5 downregulated (such as 5′-deoxy-5′-(methylthio)adenosine, 6-*O*-methylguanine, and 2-deoxy-D-ribose). Similar results were observed in the LAB fermentation of *Rosa roxburghii* Tratt juice [19], wolfberry–longan juice [35], and barley extracts [46], as well as *E. cristatum* fermentation of dark tea [47]. For instance, LAB increased the 5′-guanylic acid content in wolfberry–longan juice but decreased several purine metabolites like adenosine and guanosine [35]. Additionally, *Lc. paracasei* fermentation significantly upregulated 2′-deoxyadenosine, 2′-deoxyguanosine, and 2′-deoxyinosine in *Rosa roxburghii* Tratt juice [19].

#### 3.6.4. Lipids

Raspberries contain a lot of tiny hard seeds which are a great source of high-quality oil. Approximately, 10–25% of the oil can be recovered [48]. *Lc. paracasei* fermentation significantly changed the content of 10 lipids (3 up-regulated and 7 down-regulated) in red raspberry nectar. Three free fatty acids, namely, punicic acid (polyunsaturated fatty acid, 19.10-fold increase), octadeca-9,12,15-trienoic acid (polyunsaturated fatty acid, 8.95-fold increase), and undecylic acid (saturated fatty acid, 6.96-fold increase), were significantly upregulated. Similarly, Filannino et al. reported that the levels of mono, di-, and tri-hydroxy-octadecenoic acids in avocado nectar were enhanced by *L. plantarum* AVEF17 fermentation [49]. Interestingly, punicic acid is an “omega-5” polyunsaturated fatty acid originally found in pomegranate seed oil. To the best of our knowledge, this is the first report of its identification in red raspberry seed oil. Punicic acid is recognised as a functional substance with potential efficacy against various chronic diseases, as demonstrated in vitro and in animal models [50].

#### 3.6.5. Vitamins

Fruits are a rich source of vitamins, but they have relatively low levels of vitamin B and K [43]. It has been reported that lactic acid bacteria fermentation not only provides an acidic environment that maintain the stability of vitamins but also can synthesise water-soluble vitamins such as vitamin B [51]. *Lc. paracasei* fermentation significantly increased the concentrations of pyridoxine (VB6), biotin (VB7), and nicotinic acid (VB3) in red raspberry nectar, with elevations of 6.78, 4.99, and 2.82 times, respectively, whereas another form of VB_3_-nicotinamide in red raspberry nectar was remarkably downregulated after fermentation. This indicates that *Lc. paracasei* is a vitamin-B-producing strain and could be used to develop novel vitamin-B-enriched products. Similarly, Kaprasob et al. found that four lactic acid bacteria strains (*Lactobacillus acidophilus, Lacticaseibacillus casei, Lactiplantibacillus plantarum*, and *Leuconostoc mesenteroides*) boosted the levels of B-group vitamins (B1, B2, B3, B6, and B12) in cashew apple juice [52].

#### 3.6.6. Alkaloids

*Lc. paracasei* fermentation significantly increased the levels of 31 alkaloids in red raspberry nectar. Key examples included deoxymutaaspergillic acid (59.64-fold increase), *N*-benzoyl-2-aminoethyl-β-D-glucopyranoside (27.27-fold increase), and tryptamine (16.75-fold increase). Only three alkaloids (including *N*, *N*-cinnamoylbutanediamine, oxoassoanine *N*-oxide, and α-hydroxyquinoline) were significantly reduced. Some up-regulated alkaloids have been scientifically proven to possess specific pharmacological properties. For example, deoxymutaaspergillic acid was shown to be moderately active against MCF-7 (a breast cancer cell line) and weakly active against HepG2 cell line [53]. Indole-3-lactic acid plays a key role in anti-inflammation [54,55]. Additionally, 5-methoxy-3-indole acetic acid can activate hepatic Nrf2 and stimulate the transcription of genes involved in the cellular antioxidant response, thereby protecting the liver from acetaminophen overdose and acute ethanol toxicity [56]. These suggest that *Lc. paracasei* fermentation can greatly enhance certain beneficial alkaloids. Similar results were observed with other lactic acid bacteria [19,57].

#### 3.6.7. Phenolic Compounds

*Lc. paracasei* fermentation greatly transformed phenolic acids (13 upregulated and 4 downregulated) and flavonoids (7 upregulated and 13 downregulated) in red raspberry nectar. Some desirable phenolic acids were remarkably upregulated after fermentation. 3-(4-Hydroxyphenyl)-propionic acid (103.59-fold increase), a metabolite of procyanidin A2, has the potential to inhibit cellular oxidative stress and inflammation [58]. DL-3-phenyllactic acid (58.35-fold increase) has been proven as an ideal antimicrobial compound with broad and potent antimicrobial activity against both fungi and bacteria [59,60,61,62]. It can be converted from phenyl pyruvic acid by certain lactic acid bacteria under the action of lactic dehydrogenase [63,64]. Hydroxytyrosol (10.73-fold increase) is considered one of most powerful natural antioxidants [65], and has other beneficial effects on physic health, such as anti-inflammatory and anti-tumour activity [66,67]. Mandelic acid (2.32-fold increase) has effective anti-microbial properties [68]. Similarly, Zhao et al. reported that LAB significantly elevated the levels of certain phenolic acids (such as gallic acid, chlorogenic acid, and caffeic acid) in jujube–wolfberry composite juice and thereby improved its antioxidant capacity [45].

Most individual flavonoids in red raspberry nectar were significantly downregulated after fermentation, especially flavonoid glucosides, such as pelargonidin-3-*O*-rutinoside, delphinidin-3-*O*-rutinoside-7-*O*-glucoside, and kaempferol-3-*O*-glucoside. Montijo-Prieto et al. also observed that *L. plantarum* 748T resulted in a considerable decrease in aryl-glucosides such as quercetin-3-glucoside isomer b, protocatechuic acid 4-glucoside, luteolin-7-*O*-(2″-*O*-pentosyl)-hexoside isomer d, and kaempferol-*O*-hexoside isomer b. Likewise, Wu et al. found that the levels of cyanindin-3-glucoside and peonidin-3-glucoside in blueberry and blackberry juice dropped significantly after fermentation with *L. plantarum, Streptococcus thermophilus,* and *Bifidobacterium* [31,69]. Additionally, Qin et al. reported a progressive decrease in anthocyanins, identified as glycosides of cyanidin, pelargonidin, and delphinidin, in red raspberry juice during fermentation [18]. This might be attributed to microbial glycosidases, which hydrolyse flavonoid glucosides [70,71]. Conversely, some flavonoid glucosides were significantly upregulated, such as delphinidin-3-*O*-arabinoside, apigenin-4′-*O*-glucoside, and apigenin-7-*O*-glucoside. Similarly, Montijo-Prieto et al. reported that the concentrations of flavonoid glucosides such as luteolin-7-*O*-(2″-*O*-pentosyl)-hexoside isomer a, quercetin-diglucoside, and quercetin-3-*O*-arabinosyl-glucoside in avocado leaves were enhanced after fermentation [69].

#### 3.6.8. Terpenoids

Terpenoids are a diverse class of secondary metabolites including monoterpenoids, sesquiterpenoids, diterpenoids, triterterpenoids, tetraterpenoids, and polyterpenoids [72]. A total of eight terpenoids in red raspberry nectar were considerably affected by *Lc. paracasei*. The level of glucosyl 7-methyl-3-methyleneoctane-1,2,6,7-tetraol (monoterpenoid), shanzhiside (monoterpenoid), and ailantinol D (diterpenoid) significantly increased, while the content of 3,4-dihydroverbenalin (monoterpenoid), suavissimoside R1 (triterpene saponin), 3-hydroxy-23-oxoolean-12-en-28-oic acid (triterpene), caesalpin J (sesquiterpenoid), and (8R)-9-hydroxy-8-(hydroxymethyl)-6-methoxy-8-methylpyrano [2,3-f]chromen-2-one (terpene) in red raspberry nectar was markedly reduced after fermentation. The fermentation tended to upregulate some monoterpenoids and downregulate some higher-order terpenoids, such as triterpene and sesquiterpenoids in red raspberry nectar. This indicates that high-molecular-weight terpenoids were decomposed by *Lc. paracasei* into low-molecular-weight ones. Mandha et al. reported that most sesquiterpenes in mango juice decreased, while the total monoterpenes content remained unchanged after LAB fermentation [73].

### 3.7. KEGG Annotation and Enrichment Analysis of Differential Compounds

**Figure 4:**
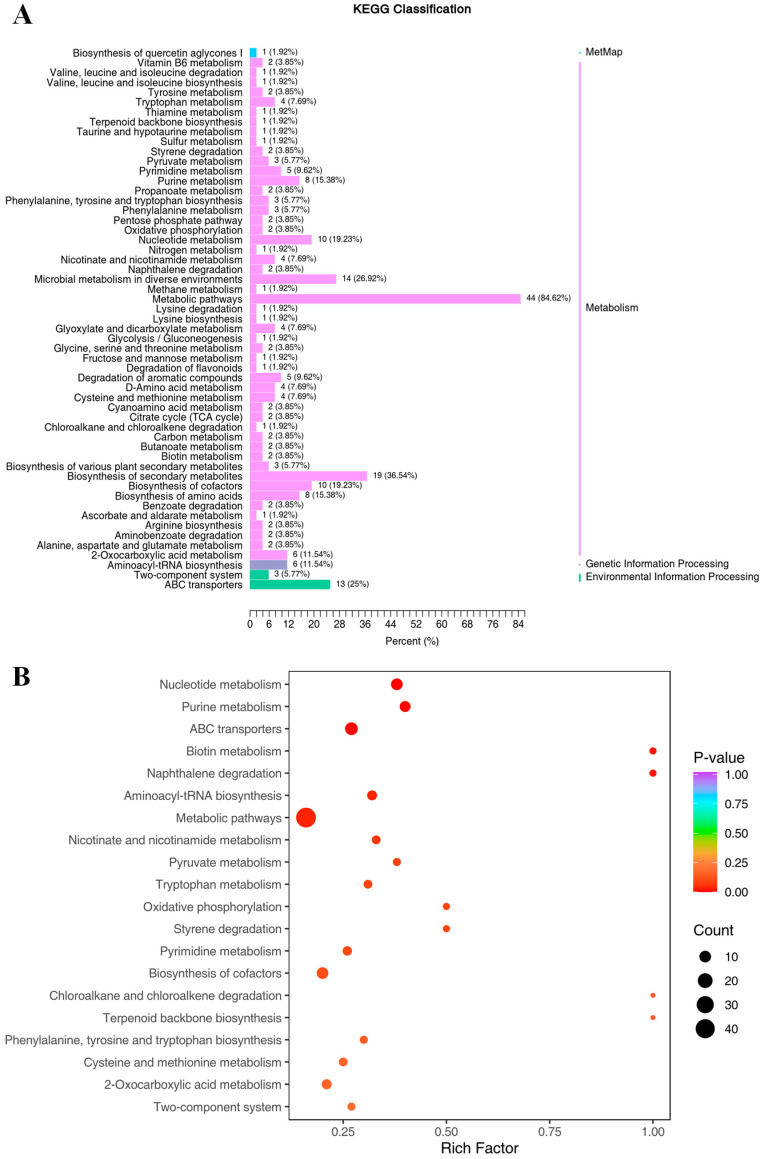
KEGG annotation and enrichment of the differential compounds: (**A**) KEGG classification for FR vs. UFR, and (**B**) KEGG enrichment map for FR vs. UFR. UFR: unfermented red raspberry nectar, FR: fermented red raspberry nectar.

Most differential compounds between FR and UFR were annotated to metabolic pathways (44 compounds) and the biosynthesis of secondary metabolites (19 compounds), as shown in Figure 4A. Furthermore, the KEGG enrichment analysis revealed that the most significantly enriched pathways were nucleotide metabolism, purine metabolism, and ABC transporters during the *Lc. paracasei* fermentation of red raspberry nectar (Figure 4B). Organic acids, amino acids, nucleotides, and their derivatives are the primary differential compounds enriched in these pathways. These results provide insights into the metabolic functions of *Lc. paracasei* and the differential compounds associated with specific pathways. However, further investigation is needed to elucidate the biotransformation mechanism, including the examination of gene expression and enzyme activity.

Similarly, the significantly enriched pathways involved in the formation of LAB-fermented *Rosa roxburghii* Tratt juice were ABC transporters, nucleotide metabolism, purine metabolism, and metabolic pathways [19], while the significantly enriched KEGG pathways involved in the LAB fermentation of wolfberry–longan juice were related to amino acid metabolism [35]. These metabolic differences are attributed to both the strain species and the substrates used.

### 3.8. The In Vitro Antioxidant Activity of UFR and FR

*Lc. paracasei* fermentation significantly improved the DPPH and hydroxyl radical scavenging abilities of red raspberry nectar by 7.26% and 6.87%, respectively, while the ABTS radical scavenging ability remained unchanged. However, its reducing power significantly decreased by 7.19% (Table 1). Similarly, LAB fermentation notably improved the DPPH and ABTS radical scavenging abilities of *Opuntia ficus*-*indica* fruit juice but did not have an impact on its ferric reducing antioxidant power [74]. In contrast, *W. anomalus* fermentation reduced the DPPH and ABTS radical scavenging abilities of black garlic juice by 5.2% and 1.4%, respectively [41]. Based on these cases, it can be concluded that fermentation affects the antioxidant capacity of substrates, influencing both the radical scavenging ability and reducing capacity, with outcomes that can be either positive or negative. The antioxidant activity is correlated with the type and content of antioxidants present. In fermentation systems, both the antioxidants originally present in the substrates and those produced by the microbes can influence the overall antioxidant capacity. Leonard et al. indicated that an enhancement in the antioxidant capacity correlates with the augmented presence of phenolic acids and other antioxidants during microbial hydrolysis reactions and metabolism [75]. Given that this study only examined two groups, it is not possible to establish direct and reliable correlations between specific antioxidants and the antioxidant capacity. Future research involving a broader range of groups helps to identify key antioxidants and their contributions to antioxidant activity.

## 4. Conclusions

*Lc. paracasei* FBKL 1.0328 fermentation significantly modified the nutrients and phytochemicals of red raspberry nectar, resulting in 261 significant differential compounds (198 upregulated and 63 downregulated). Amino acids and derivatives (*n* = 113), phenolic compounds (*n* = 37), and alkaloids (*n* = 34) were the three largest groups of differential components between the unfermented red raspberry nectar and the fermented one. Certain desirable nutrients (e.g., essential amino acids, lactic acid, and vitamin B) and bioactive compounds (e.g., 3-(4-hydroxyphenyl)-propionic acid, DL-3-phenyllactic acid, and ndole-3-lactic acid) were notably upregulated. Simultaneously, the fermentation significantly enhanced the DPPH and OH radical scavenging abilities of red raspberry nectar. Furthermore, the KEGG enrichment analysis uncovered that nucleotide metabolism, purine metabolism, and ABC transporters contributed significantly to the new non-volatile composition profile of fermented red raspberry nectar. These findings provide a sound reference for the development of fermented red raspberry products.

## Figures and Tables

**Table 1:** The effects of *Lc. paracasei* fermentation on the basic physicochemical parameters of red raspberry nectar.

	UFR	FR
Viable cells (Log CFU/mL)	-	9.84 ± 0.03 **
L*	25.74 ± 0.54	22.51 ± 0.43 *
a*	36.83 ± 1.17	38.27 ± 1.59
b*	12.11 ± 1.78	11.67 ± 1.89
Total soluble solids (%)	6.10 ± 0.00	5.83 ± 0.07 *
Total sugar (μg/mL)	463.38 ± 5.30	458.49 ± 8.62
Reducing sugar (μg/mL)	278.62 ± 2.27	297.63 ± 9.18
pH	3.25 ± 0.00	3.12 ± 0.00 **
Total acidity (g/L)	9.92 ± 0.00	12.24 ± 0.09 **
Total polyphenols (μg/mL)	346.39 ± 4.14	336.50 ± 7.24
Total flavonoids (μg/mL)	482.28 ± 3.39	446.84 ± 3.33 **
DPPH radical scavenging rate (%)	64.69 ± 0.60	69.37 ± 0.54 *
ABTS radical scavenging rate (%)	93.17 ± 0.39	93.09 ± 0.49
Hydroxyl radical scavenging rate (%)	89.25 ± 1.06	95.38 ± 1.13 *
Reducing power (%)	86.34 ± 1.40	80.13 ± 1.37 **

*: *p* < 0.05; **: *p* < 0.01; “-”: none; UFR: the unfermented red raspberry nectar; FR: the fermented red raspberry nectar.

**Table 2:** The top 50 differential metabolites between FR and UFR.

Category	Compounds	Ionization Model	Precursor Ions (Da)	Product Ions (Da)	Formula	VIP	FC	Type
Amino acids and derivatives (20)	Asp-Phe-Arg	[M+H]^+^	437.21	156.08	C_19_H_28_N_6_O_6_	1.51	108.26	up
	Cyclo(L-Phe-trans-4-hydroxy-L-Pro)	[M+H]^+^	261.12	120.08	C_14_H_16_N_2_O_3_	1.49	48.71	up
	*N*-(1-deoxy-1-fructosyl)phenylalanine	[M-H]^-^	326.12	164.07	C_15_H_21_NO_7_	1.54	36.89	up
	Gly-Gly-Gln	[M+H]^+^	261.12	136.07	C_9_H_16_N_4_O_5_	1.52	36.62	up
	Cyclo(L-tyrosyl-D-proline)	[M+H]^+^	261.12	136.08	C_14_H_16_N_2_O_3_	1.53	33.71	up
	Cyclo (L-prolyl-L-tyrosine)	[M+H]^+^	261.12	136.08	C_14_H_16_N_2_O_3_	1.53	32.12	up
	Cyclo(D-Val-L-Pro)	[M+H]^+^	197.13	70.07	C_10_H_16_N_2_O_2_	1.54	30.74	up
	Gly-Val-Val	[M+H]^+^	274.18	129.1	C_12_H_23_N_3_O_4_	1.54	23.75	up
	Pro-Asn-Leu	[M+H]^+^	343.2	70.07	C_15_H_26_N_4_O_5_	1.46	22.36	up
	Cyclo(Pro-Val)	[M+H]^+^	197.13	72.08	C_10_H_16_N_2_O_2_	1.54	22.12	up
	Tyr-Pro-Lys	[M+H]^+^	407.23	323.2	C_20_H_30_N_4_O_5_	1.48	21.70	up
	Pro-Gln-Val	[M+H]^+^	343.2	155.09	C_15_H_26_N_4_O_5_	1.46	18.76	up
	*N*-(1-deoxy-1-fructosyl)leucine	[M-H]^-^	292.14	130.09	C_12_H_23_NO_7_	1.54	16.12	up
	Cyclo(L-Ala-L-Pro)	[M+H]^+^	169.1	70.07	C_8_H_12_N_2_O_2_	1.54	15.34	up
	Gly-Leu-Val	[M+H]^+^	288.19	143.12	C_13_H_25_N_3_O_4_	1.53	13.50	up
	L-alanyl-L-leucine	[M+H]^+^	203.14	86.1	C_9_H_18_N_2_O_3_	1.54	13.26	up
	Ile-Glu-Val	[M+H]^+^	360.21	229.12	C_16_H_29_N_3_O_6_	1.54	13.17	up
	L-seryl-L-isoleucine	[M+H]^+^	219.13	60.04	C_9_H_18_N_2_O_4_	1.42	12.46	up
	Cyclo(Pro-Phe)	[M+H]^+^	245.13	120.08	C_14_H_16_N_2_O_2_	1.52	12.14	up
	Ala-Ile-Asn	[M+H]^+^	317.18	120.08	C_13_H_24_N_4_O_5_	1.54	11.89	up
Phenolic acids (8)	2-Hydroxy-3-phenylpropanoic acid	[M-H]^-^	165.06	103.06	C_9_H_10_O_3_	1.54	257.27	up
	Tropic acid	[M-H]^-^	165.06	103.06	C_9_H_10_O_3_	1.54	228.82	up
	3-(4-Hydroxyphenyl)-propionic acid	[M-H]^-^	165.06	119.05	C_9_H_10_O_3_	1.54	103.59	up
	DL-3-phenyllactic acid	[M-H]^-^	165.06	119.05	C_9_H_10_O_3_	1.54	58.35	up
	Methyl 2,4-dihydroxyphenylacetate	[M-H]^-^	181.05	135.04	C_9_H_10_O_4_	1.54	53.85	up
	2-Hydroxy-3-(4-hydroxyphenyl)propanoic acid	[M-H]^-^	181.05	135.04	C_9_H_10_O_4_	1.54	45.35	up
	4-Hydroxyphenyllactic acid	[M-H]^-^	181.05	135.04	C_9_H_10_O_4_	1.54	45.13	up
	10-Hydroxymajoroside	[M-H]^-^	403.12	241.07	C_17_H_24_O_11_	1.54	12.81	up
Alkaloids (7)	Deoxymutaaspergillic acid	[M+H]^+^	211.14	70.06	C_11_H_18_N_2_O_2_	1.54	59.64	up
	*N*-benzoyl-2-aminoethyl-β-D-glucopyranoside	[M+H]^+^	328.13	310.12	C_15_H_21_NO_7_	1.54	27.27	up
	Tryptamine	[M+H]^+^	161.11	144.08	C_10_H_12_N_2_	1.53	16.75	up
	1,2-Dihydro-13-norgalanthamine	[M+H]^+^	276.16	120.08	C_16_H_21_NO_3_	1.54	12.81	up
	(22R,25R)-16β-*H*-22a-*N*-spirosol-3β-ol-5-ene-3-*O*-rhamnosyl(1→2)[rhamnosyl(1→4)]glucoside	[M+H]^+^	868.5	868.5	C_45_H_73_NO_15_	1.54	12.64	up
	*N*-feruloyltyramine 4′-glucoside	[M+H]^+^	476.2	177.05	C_24_H_29_NO_9_	1.51	12.42	up
	2-(Acetylamino)-3-phenyl-2-propenoic acid	[M+H]^+^	206.08	118.07	C_11_H_11_NO_3_	1.54	12.41	up
Organic acids (6)	2-Hydroxy-4-methylpentanoic acid	[M-H]^-^	131.07	85.07	C_6_H_12_O_3_	1.54	61.26	up
	2-Hydroxyisocaproic acid	[M-H]^-^	131.07	85.07	C_6_H_12_O_3_	1.54	54.26	up
	Muconic acid	[M-H]^-^	141.02	59.01	C_6_H_6_O_4_	1.52	20.69	up
	L-Tartaric acid	[M-H]^-^	149.01	87.01	C_4_H_6_O_6_	1.54	15.80	up
	L-Lactic acid	[M-H]^-^	89.02	71.02	C_3_H_6_O_3_	1.53	13.29	up
	Oxalic acid	[M-H]^-^	88.99	70.98	C_2_H_2_O_4_	1.53	13.07	up
Nucleotides and derivatives (3)	2′-Deoxyadenosine	[M+H]^+^	252.11	136.06	C_10_H_13_N_5_O_3_	1.51	173.83	up
	Inosine	[M+H]^+^	269.09	137.05	C_10_H_12_N_4_O_5_	1.54	12.33	up
	9-(Arabinosyl)hypoxanthine	[M-H]^-^	267.07	135.03	C_10_H_12_N_4_O_5_	1.53	12.18	up
Lignans and Coumarins (1)	Dihydrosesamin	[M+H]^+^	357.13	307.09	C_20_H_20_O_6_	1.44	0.07	down
Lipids (1)	Punicic acid	[M+H]^+^	279.23	95.09	C_18_H_30_O_2_	1.45	19.10	up
Others (4)	2,6-Dimethoxybenzaldehyde	[M-H]^-^	165.06	119.05	C_9_H_10_O_3_	1.54	57.99	up
	3-Ethyl-7-hydroxyphthalide	[M+H]^+^	179.07	119.05	C_10_H_10_O_3_	1.41	22.87	up
	2,3-Dihydroxypropanal	[M-H]^-^	89.02	71.02	C_3_H_6_O_3_	1.53	12.48	up
	Nicotinamide	[M+H]^+^	123.06	80.05	C_6_H_6_N_2_O	1.54	0.01	down

VIP: Variable Importance in Projection; FC: Fold Change; FR: the fermented red raspberry nectar; UFR: the unfermented red raspberry nectar.

## Data Availability

The original contributions presented in the study are included in the article/Supplementary Material, further inquiries can be directed to the corresponding author.

## Supplementary Materials

The following supporting information can be downloaded at: https://www.mdpi.com/article/10.3390/foods13223666/s1, Figure S1:The total ion current (TIC) of samples including quality control (QC); Figure S2: Coefficient of Variation (CV) distribution diagram of each group of samples.
