# Supplementary material for: Nutrients, Phytochemicals, and Antioxidant Capacity of Red Raspberry Nectar Fermented with Lacticaseibacillus paracasei
Source: Foods. 2024 Nov 18;13(22):3666. doi: 10.3390/foods13223666 (PMC11593764; doi:10.3390/foods13223666)
Supplement: Supplementary file 1 [file foods-13-03666-s001.zip › Supplementary Fig.S1.pdf]

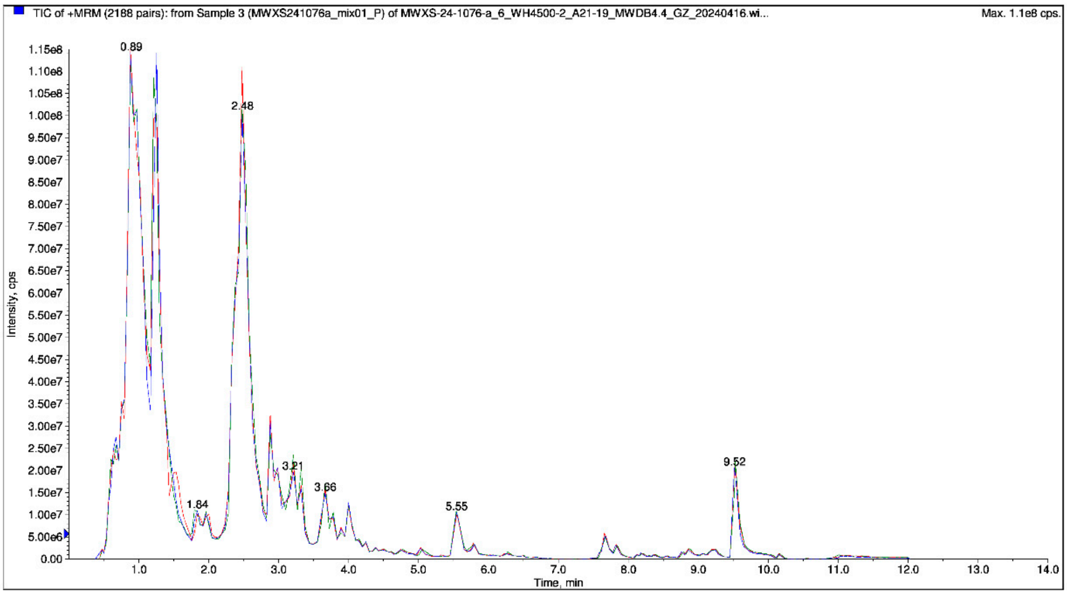

(A)

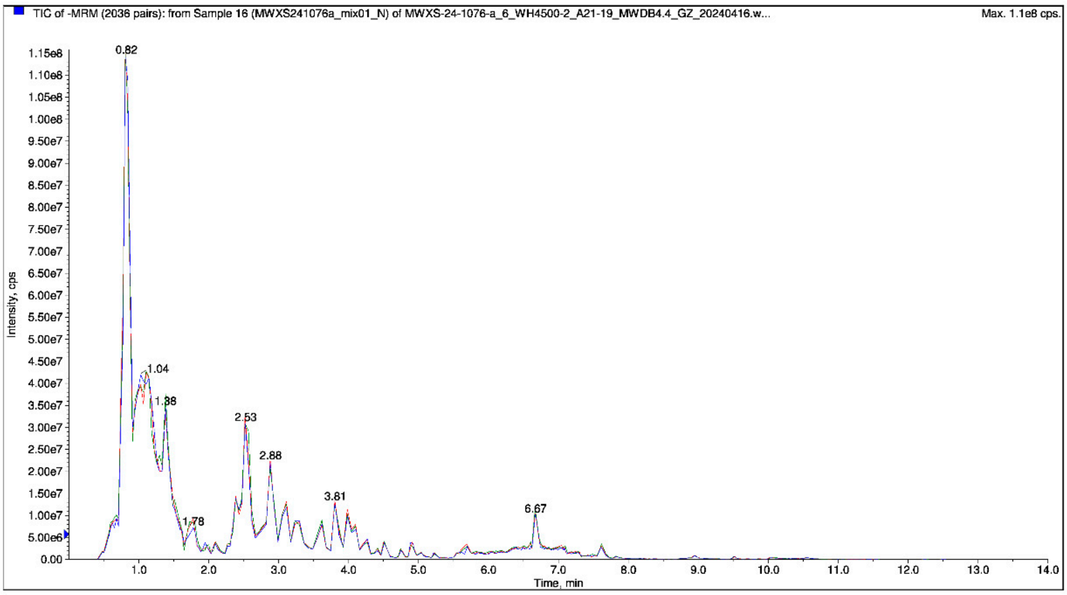

(B)

Fig.S1. The total ion current (TIC) of samples including QC. QC: quality control; (A) positive ionization mode; (B) negative ionization mode.
