# Supplementary material for: Nutrients, Phytochemicals, and Antioxidant Capacity of Red Raspberry Nectar Fermented with Lacticaseibacillus paracasei
Source: Foods. 2024 Nov 18;13(22):3666. doi: 10.3390/foods13223666 (PMC11593764; doi:10.3390/foods13223666)
Supplement: Supplementary file 1 [file foods-13-03666-s001.zip › Supplementary Fig.S2.pdf]

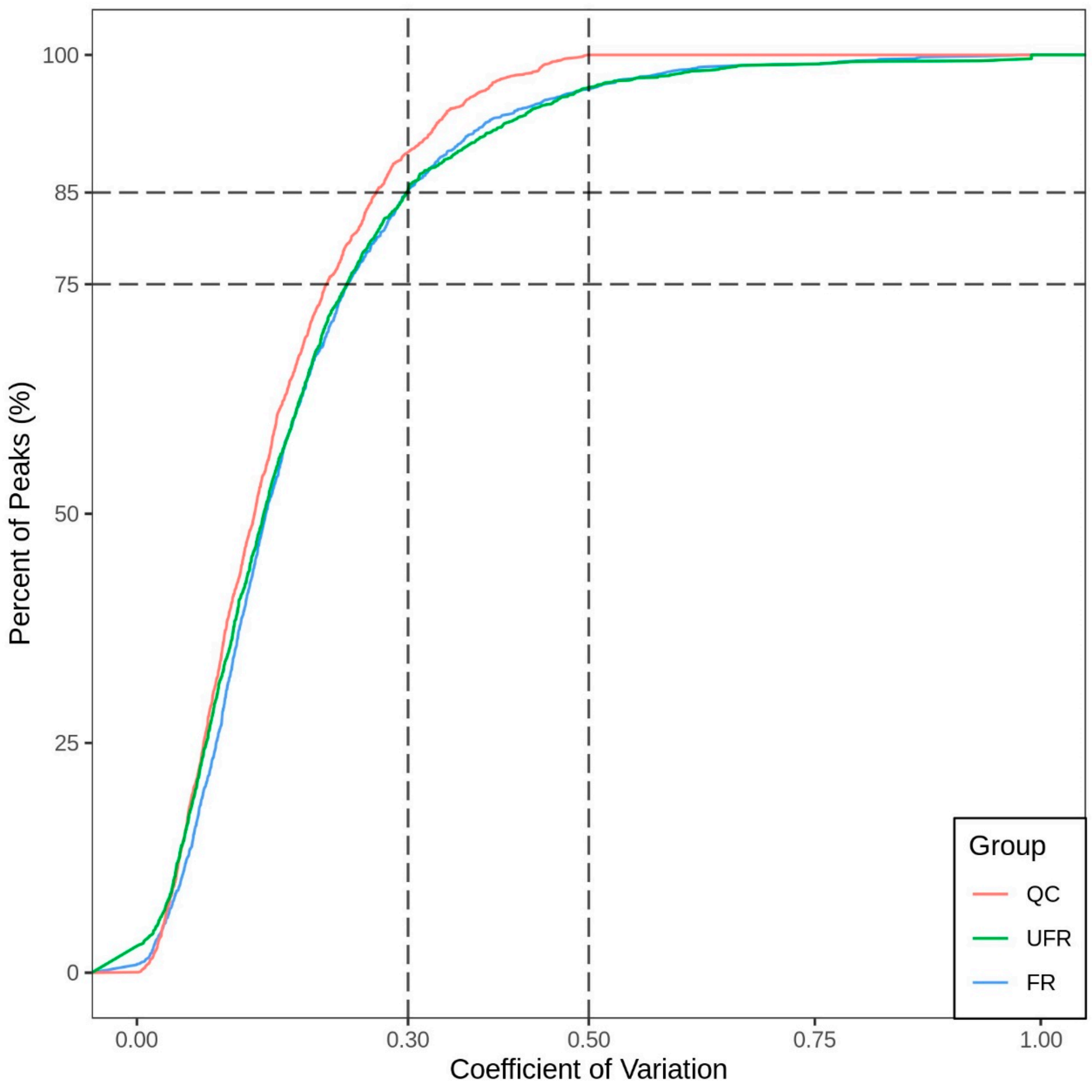

Fig.S2. CV (Coefficient of Variation) distribution diagram of each group of samples. QC: quality control; UFR: unfermented red raspberry pulp; FR: fermented red raspberry pulp.
